# *In Vitro* Anti-Inflammatory and *In Vivo* Antiarthritic Activities of Aqueous and Ethanolic Extracts of *Dissotis thollonii* Cogn. (Melastomataceae) in Rats

**DOI:** 10.1155/2019/3612481

**Published:** 2019-11-15

**Authors:** Stephanie Flore Djuichou Nguemnang, Eric Gonzal Tsafack, Marius Mbiantcha, Ateufack Gilbert, Albert Donatien Atsamo, William Yousseu Nana, Vanessa Matah Marthe Mba, Carine Flore Adjouzem

**Affiliations:** ^1^Laboratory of Animal Physiology and Phytopharmacology, Department of Animal Biology, Faculty of Science, University of Dschang, PO. Box 67, Dschang, Cameroon; ^2^Laboratory of Animal Physiology, Faculty of Science, University of Yaounde I, PO Box 812, Yaoundé, Cameroon

## Abstract

*Dissotis thollonii* Cogn. (Melastomataceae) is a tropical plant widely used in traditional Cameroonian medicine to relieve and treat many pathologies. It is widespread in the western region where it is used to treat typhoid fever, gastrointestinal disorders, and inflammatory diseases. The purpose of this study is to scientifically demonstrate the anti-inflammatory and antiarthritic properties of the aqueous and ethanolic extracts of the leaves of *Dissotis thollonii*. The anti-inflammatory properties were evaluated in vitro by inhibition tests for cyclooxygenase, 5-lipoxygenase, protein denaturation, extracellular ROS production, and cell proliferation; while antiarthritic properties were evaluated in vivo in rats using the zymosan A-induced monoarthritis test and the CFA-induced polyarthritis model. This study shows that aqueous and ethanolic extracts at a concentration of 1000 *μ*g/ml inhibit the activity of cyclooxygenase (47.07% and 63.36%) and 5-lipoxygenase (66.79% and 77.7%) and protein denaturation (42.51% and 44.44%). Similarly, both extracts inhibited extracellular ROS production (IC_50_ = 5.74 *μ*g/ml and 2.96 *μ*g/ml for polymorphonuclear leukocytes, 7.47 *μ*g/ml and 3.28 *μ*g ml for peritoneal macrophages of mouse) and cell proliferation (IC_50_ = 16.89 *μ*g/ml and 3.29 *μ*g/ml). At a dose of 500 mg/kg, aqueous and ethanolic extracts significantly reduce edema induced by zymosan A (69.30% and 81.80%) and CFA (71.85% and 79.03%). At the same dose, both extracts decreased sensitivity to mechanical hyperalgesia with 69.00% and 70.35% inhibition, respectively. Systemic and histological analyzes show that both extracts maintain the studied parameters very close to normal and greatly restored the normal architecture of the joint in animals. *Dissotis thollonii* would therefore be a very promising source for the treatment of inflammatory diseases.

## 1. Introduction

Rheumatoid arthritis (RA) is a chronic, disabling, and progressive autoimmune disease in which chronic proliferative synovitis and synovial inflammation arde observed with significant bone destruction and cartilage destruction resulting in significant joint damage and reduced functionality [1–3]. This pathology can evolve very quickly in an individual and affect several parts of the body that become inflamed or extremely painful particularly affecting the elderly, but also individuals with degenerative bone disorder or immune system dysfunction [4]. This pathology, which can also occur as a result of the immune system attacking the synovial membrane, is accompanied by swelling, stiffness, pain, and a reduction or loss of joint function [4]. During the establishment and development of rheumatoid arthritis, many inflammatory mediators play a key role in bone destruction and inflammation of the synovial membrane, including tumor necrosis factor (TNF-*α*), interleukin-1*β*, interleukin-6, nitric oxide (NO), prostaglandins, reactive oxygen species (ROS), platelet-activating factor, leukotrienes, enzymes (lipoxygenases, cyclooxygenases (COX-1 and COX-2), and phospholipases) [5, 6].

Animal models of zymosan A-induced monoarthritis and Freund's complete adjuvant-induced polyarthritis (CFA) are widely used in research for the activity of many pharmacological substances on rheumatoid arthritis. In fact, the injection of zymosan into the knee joint of the rats causes erosive synovitis with increased vascular permeability, neutrophil infiltration, and formation of edemas and exudates in the acute phase, and then in the chronic phase, infiltration of macrophages and lymphocytes, pannus formation, and fibroblastic reaction characteristic of chronic rheumatoid synovitis [7, 8]. CFA is an immunogenic adjuvant consisting of a suspension of *Mycobacterium tuberculosum* or *Mycobacterium butyricum* killed by heat. When injected at the base of the animal's tail, it causes the development of polyarthritis that evolves in a two-phase cycle of time: the first phase appears in a few hours and disappears after 3 to 5 days and manifests itself by an acute local inflammatory reaction, and then the second phase appears after two weeks and corresponds to a chronic systemic reaction [9–11]. This polyarthritis is not primarily aimed at the knee joint, and it can affect the general state of the animal body; it is a real systemic disease resulting in inflammation of the distal joints of the limbs, vertebrae, lesions of the genitourinary tract, gastrointestinal tract, eyes, nose, ears, skin, and anorexia accompanied by significant weight loss [9, 11]. In addition, the pathology will persist, and other symptoms will appear, namely, joint deformity, synovitis, synovial hyperplasia, capsular fibrosis, angiogenesis, pannus formation, cartilage destruction, bone erosion, inflammation of the bone marrow, resorption of bone matrix, and ankylosis [12].

The severity and persistence of rheumatoid arthritis require long-term management with anti-inflammatory drugs. Nevertheless, these anti-inflammatory drugs have for the most part risks of toxicity for long-term use, which seriously limits their use. Current research in the management of rheumatoid arthritis is turning to a new generation of substances capable of selectively inhibiting TNF alpha and/or cyclooxygenase (COX-2) and having no major side effects [13]. Recent interest in alternative treatments for arthritis favors the use of traditional medicine although scientific evidence of efficacy for most cases is lacking. Nevertheless, several herbs, used in a care program and a very effective preventive medicine, can act individually and/or in synergy to reduce chronic joint inflammation (osteoarthritis and/or rheumatoid arthritis) [14–16]. To reach the total health care coverage of the world's population, traditional medicine is considered by WHO to be the most effective means since about 25% of modern prescription drugs are more or less obtained from plants [17, 18].

Comprising about 163 genera, the family of Melastomataceae which are mainly pantropical plants include more than 4,300 species so many of them are known for their effectiveness in traditional medicine as antihepatitic, antihypertensive, anti-inflammatory, antihyperglycaemic, antioxidant, hemostatic, and antidiarrheal [19–24]. *Dissotis thollonii* (*D. thollonii*) is one of many species of the family of Melastomataceae used in traditional medicine in Cameroon to treat typhoid fever, gastrointestinal disorders, inflammatory diseases, and sinusitis [25–27]. The leaves are recommended for the treatment of ulcers and gastrointestinal disorders. A previous study showed that *D. thollonii* significantly inhibited fluid accumulation in intestine induced by prostaglandin E2 [28]. Based on recent work by Ateufack et al. [29], this plant has antidiarrheic and antibacterial properties and then has several secondary metabolites including tannins, flavonoids, sterols, anthraquinones, phenols, and polyphenols. In addition, the work of TadjouaTchoumbou et al. [24] showed that this plant significantly inhibited leukocyte migration in peritoneal fluid, intracellular ROS production, proliferation of Hela cell lines, and TNF-*α* production. Tala et al. [27] showed that aqueous and ethanolic extracts were devoid of toxicity after 28 days of daily treatment. Similarly, Nono et al. [30] showed the antimicrobial and antioxidant properties of this plant. Several compounds have already been isolated from this plant, among which 3,3′-diomethylellagic acid 4′-O-*β*-D-xylopyranoside, 3-4′-O-*β*-D- arabinopyranoside, casuarinine, betulinic acid, *β*-sitosterol-3-O-D-glucopyranosyl-6′-mirystate, cellobiosylsterol, *β*-sitosterol, *β*-sitosterol-3-O-*β*-D-glucopyranoside, arjunolic acid, 3,3′-diomethylellagic acid, ellagic acid, and 3,3′-diomethylellagic acid 4′-O-*β*-D-glucopyranoside [30]. Although this plant is traditionally used to relieve many disorders of the body, no information or scientific report to our knowledge has been found in the literature relative to its antiarthritic properties. In our continuous search for bioactive extracts from plants used in traditional Cameroonian medicine [31, 32], and in order to support and improve the traditional use of *D. thollonii*, we undertook to carry out the present study on in vitro anti-inflammatory activities and in vivo antiarthritic activities of the leave extracts of *D. thollonii*.

## 2. Materials and Methods

### 2.1. Plant Material and Extraction

The plant material, referenced to the national herbarium of Cameroon under the number N° 133292/SRF Cam, in the name of *D. thollonii* (Melastomotaceae) was used in this study. The fresh leaves were harvested in the town of Dschang (western Cameroon), dried in the shade, and then crushed into a fine powder. In order to prepare the aqueous extract, 500 g of powder was mixed into 500 ml of distilled water during 72 hours and filtrated (Whatman paper No. 4); the filtrate obtained was evaporated at 40°C to give the aqueous extract (8.2% yield). The same weight of dried powder plant was mixed into 500 ml of ethanol for 72 hours and then filtered. The filtrate was concentrated with a rotary evaporator set at 96°C to give the ethanolic extract with 9.6% yield.

### 2.2. Phytochemical Assay of *D. thollonii* Extracts

The different extracts were subjected to chemical screening in order to detect the presence of the main groups of compounds following the principles stated by Matos [33].

### 2.3. Triterpenes and Steroids: Lieberman–Burchard Test

In a tube containing 3 ml of MeOH, 0.1 g of extracts was dissolved, and then 0.2 ml of each of the following reagents was added: chloroform, glacial acetic anhydride, and concentrated H_2_SO_4_. The mixture was observed to look for the appearance of the greenish-blue or purple-pink color characteristics of the presence of sterols and triterpenes, respectively.

### 2.4. Phenols

The tube contained 3 ml of ethanol; 0.1 g of extracts was dissolved, and then three drops of 10% iron III chloride were added. The solution was then observed to observe for the appearance of the blue-violet or greenish coloration which characterizes the presence of the phenols.

### 2.5. Tannins

5 ml of MeOH was introduced in the tube, and 0.1 g of extracts was dissolved. The solution was then added with 5 drops of 0.5% sulfuric acid, and the mixture was observed to detect the green or blue-black color, indicating the presence of tannins.

### 2.6. Flavonoids: Shinoda Test

In a tube containing 3 ml of MeOH, 0.1 g of extracts was dissolved, and the mixture was then treated with 0.05 g of magnesium chloride chips and 3 drops of concentrated H_2_SO_4_. The flavonoids were highlighted by the appearance of the following colorings: orange for flavones, red for xanthones, and pink for flavonols.

### 2.7. Anthocyanings

In a test tube containing 0.1 g of extracts, 5 drops of concentrated hydrochloric acid were introduced. The solution was then observed to look for the appearance of red color indicating the presence of anthocyanins.

### 2.8. Saponins

The presence of saponins is generally materialized by the formation of a stable foam after stirring a solution. Thus, a solution of 5 ml of distilled water and 5 ml of each extract was vigorously shaken to check for the presence or absence of saponins in each extract of our plant.

### 2.9. Anthraquinone

The presence of free anthraquinones and/or anthraquinone derivatives in a mixture is indicated by a pink, violet, or red coloration in the lower phase (ammoniacal phase) of the mixture after stirring. Thus, two methods made it possible to verify the presence of this class of compound in our various extracts:

- Stirring a solution prepared in the following manner: extract (3 ml) and benzene (3 ml), followed by filtration and then 5 ml ammonia (10%) in the filtrate.
- A mixture of extract (3 ml) and sulfuric acid (3 ml) is boiled and filtered while hot and benzene (3 ml) is added to the filtrate followed by stirring. After separation of the benzene layer, ammonia prepared at 10% (3 ml) was added.

## 3. *In Vitro* Anti-Inflammatory Assays

### 3.1. Inhibition of Protein Denaturation

To evaluate the anti-inflammatory effects of the extracts, the protocol described by Padmanabhan and Jangle [34] and Elias and Rao [35] was used with small modifications. A volume of 1 ml of extracts (aqueous and ethanolic) or of diclofenac sodium at different concentrations (100, 200, 500, and 1000 *μ*g/ml) was homogenised with 1 ml of aqueous solution of bovine serum albumin (5%) and incubated at 27°C for 15 minutes. The mixture of distilled water and BSA constituted the control tube. Denaturation of the proteins was caused by placing the mixture in a water bath for 10 minutes at 70°C. The mixture was cooling inside the ambient room temperature, and the activity each mixture was measured at 660 nm. Each test was done three times. The following formula was used to calculated inhibition percentage:

(1) $$\begin{array}{c} \%\text{ inhibition}=\frac{\text{absorbance of control}-\text{absorbance of sample}}{\text{absorbance of control}}\times 100\text{.} \end{array}$$

## 4. Assay of Cyclooxygenase and 5-Lipoxygenase Inhibition

### 4.1. Lymphocyte Culture Preparation

RPMI 1640 (HIMEDIA) added with inactivated fetal calf serum, penicillin, and streptomycin was used for culture of human peripheral lymphocytes, and cell proliferation was induced by phytohemagglutinin (HIMEDIA). After filtration (using 0.2 micron cellulose acetate, Sartorios), plasma was added (1 × 10^6^ cells/ml) and incubated for 72 hours, and the culture was activated by the addition of lipopolysaccharide (1 *μ*l) and incubated again for 24 hours. Extracts (aqueous and ethanolic) and ibuprofen were added at a final concentration of 100, 200, 500, and 1000 *μ*g/ml, incubated for 24 hours, and centrifuged for sedimentation at 6000 rpm for 10 min. After removal of the supernatant, cell lysis buffer was added (50 *μ*l), and the mixture was again centrifuged at 6000 rpm for 10 min. The anti-inflammatory test was performed according to the method used by Viji and Helen [36].

### 4.2. Assay of Cyclooxygenase

A mixture of glutathione, tris-HCl buffer, enzyme, and hemoglobin was used to make the assays. After addition of arachidonic acid and TCA (10% in 1N HCl, 0.2 ml), the mixture was incubated at 37°C (20 minutes). The TBA (0.2 ml) was added to the contents which were then heated (for 20 minutes in boiling water), and after cooling, the mixture was centrifuged (1000 rpm, 3 min) and the supernatant was used to measure COX activity at 632 nm [36].

### 4.3. Assay of 5-Lipoxygenase

In 4 ml of nonoxygenated water, linoleic acid (70 mg) and an equal weight of interpolation were dissolved and pipetted, followed by sodium hydroxide (0.5 N) and water without oxygen (25 mL) was added. The final solution was divided into small portions of 0.5 ml each and rinsed with nitrogen and frozen. A quartz cuvette (25°C) optical path of 1 cm made it possible to carry out the reaction. The OD measured at 234 nm was performed with a mixture of tris buffer (2.75 ml, pH 7.4), sodium linoleate (0.2 ml), and enzyme (50 ml) [36]. The following formula was served to determine percent inhibition:

(2) $$\begin{array}{c} \%\text{ inhibition}=\left[\frac{\left\{\text{abs control}-\text{abs sample}\right\}}{\text{abs control}}\right]\times 100. \end{array}$$

### 4.4. Chemiluminescence Assay

The human blood samples used in this work were received from a donor following the procedure accepted by the Independent Ethics Committee, ICCBS, University of Karachi, No  : ICCBS/IEC-008-BC-2015/Protocol/1.0. The blood donors were informed that it should be used for an experimental study.

### 4.5. Isolation of Human Polymorphoneutrophils (PMNs)

Aseptically, a volume of 10 ml of venous blood was taken from a very healthy 33-year-old volunteer donor and then introduced into a tube with anticoagulant (heparin). Neutrophils were isolated following the protocol described by hypoflat Ficoll density gradient centrifugation [37]. For this, in an empty tube of 45 ml, the whole blood, HBSS, and LSM (lymphocyte separation medium) were introduced at equal volume, and after 30 min latency, the supernatant of this mixture was then removed and introduced into a 15 ml tube previously containing 5 ml of LSM; then, the tube was centrifuged at 400 g for 20 minutes (room temperature). After removal of the supernatant, 1 ml of distilled water was introduced into the tube (for lysis of red blood cells); after a duration of 1 minute, the HBSS (2x) (1 ml) was also introduced (to stop the lysis). After adding 5 ml of HBSS again, the tube was centrifuged for 10 minutes at 4°C (300 g), and 1 ml of HBSS was added after removal of the supernatant and the tube was kept in ice. The trypan blue technique was used to assess viability while the hemocytometer counted the cells. For each test, the cell concentration used was 1 × 10^6^ cells/ml.

### 4.6. Peritoneal Macrophage Isolation from Mice

One milliliter of FBS was injected (intraperitoneally) into NMRI mice weighing an average of 22 g, and these animals were kept and observed for 3 days; then, they underwent cervical dislocation for sacrifice. The RPMI medium (10%, 10 ml) was introduced into the peritoneal cavity, after 2 minutes of massage, the skin of the abdomen was removed, and the peritoneal cavity was exposed. Using a syringe, the RPMI medium injected into the peritoneum and containing the macrophages was removed, introduced into a tube, centrifuged (400 g, 20 minutes, 4°C), the supernatant was removed, 5 ml incomplete RPMI was supplemented, the tube was further centrifuged (300 g, 10 minutes, 4°C), and then RPMI/HBSS (1 ml) was supplemented. The trypan blue technique was used to assess viability, while the hemocytometer counted the cells. For each test, the cell concentration used was 1 × 10^6^ cells/ml [38, 39].

For the chemiluminescence assay, the modified protocol of Mbiantcha et al. [40] was used. In white plates (96 wells), 25 *μ*l of PMNs cells, whole blood or macrophages, 25 *μ*l of extracts (aqueous or ethanolic), or ibuprofen were mixed. While the well without extracts represent the control and received only cells and HBSS^++^. After 20 minutes of incubation, each well obtained 25 *μ*l of luminol and 25 *μ*l of PMA, which made it possible to obtain a total volume of 100 *μ*l in each well. After completing this test, the results were expressed in RLU (relative light units), and the following formula allowed to calculate the percentage of inhibition:

(3) $$\begin{array}{c} \text{inhibition}\left(\%\right)=\frac{\left(\text{RLUcontrol}-\text{RLUsample}\right)}{\text{RLUcontrol}}\times 100. \end{array}$$

### 4.7. T-Cell Proliferation Assay [41]

96-well white plates were used for this test. In each well, were introduced the extracts (2, 10 and 50 *μ*g/ml), prednisolone (diluted in RPMI (5%)), 50 *μ*l of T lymphocytes at a concentration of 2 × 10^6^ cells/ml, 50 *μ*l of PHAL at a concentration of 7.5 *μ*g/ml (phytohemagglutinin-L). Wells considered as negative controls received only 550 *μ*l of cells and 150 *μ*l of RPMI (5%), whereas those considered as positive controls received 50 *μ*l of cells, 50 *μ*l of PHA, and 100 *μ*l of RPMI (5%). The plates were then incubated (3 days, 37°C, CO_2_ (5%)), 25 *μ*l of 0.5 *μ*Ci/well (methyl 7 3H) thymidine was used to pulse the cultures, followed by a second incubation for 18 hours, and then the cells were harvested (using a fiberglass filter). The level of thymidine integrated in the cells was determined using a counter (LS65000 liquid scintillation). The following formula allowed to calculate the percentage of inhibition using CPM (counts per minute):

(4) $$\begin{array}{c} \text{inhibitory activity}\left(\%\right)=\frac{\left(\text{CPMcontrol}-\text{CPMsample}\right)}{\text{CPMcontrol}}\times 100. \end{array}$$

## 5. *In Vivo* Antiarthritis Assays

### 5.1. Animals

Female Wistar rats (3 to 4 months old and 150 to 200 g) were used for these tests. The animals were breeded from the faculty of science (laboratory of animal physiology and phytopharmacology) of the University of Dschang (Cameroon). They were raised under normal conditions (19–23°C, 12 hour light) with water and access without diet.

The experimental procedures have been approved by the local Ethics Committee and are in accordance with the guidelines for the study of pain in awake animals, published by the NIH (publication no. 85-23, “Principles of Animal Protection,” Laboratory, and Study of Pain, Ministry of Scientific Research and Technology, which adopted the European Union Guidelines on Animal Care and Experimentation (EWC 86/609).

### 5.2. Treatment Regimen

Animals were grouped by their weight into different cages, and in each group, they were identified by the tail with a number using a marker pen. In each test, 42 female rats were divided into 7 groups (6 rats each): group 1 (healthy control) received no treatment and no injection of zymosan A (Sigma Chemical Co., St. Louis, Missouri, USA) or CFA (Sigma Chemical Co., St. Louis, Missouri, USA), group 2 (arthritic control group) received the solution of DMSO (5%) + PBS with injection of zymosan A or CFA, group 3 (positive control) received diclofenac (5 mg/kg) with injection of zymosan A or CFA, groups 4 and 5 received the aqueous extract of *D. thollonii* (250 and 500 mg/kg) with injection of zymosan A or CFA, and groups 6 and 7 received the ethanolic extract of *D. thollonii* (250 and 500 mg/kg) with injection of zymosan A or CFA. All treatments were administered orally 1 hour before the induction of zymosan A or CFA (day 0), and then the animals were treated daily for 5 days for zymosan A-induced monoarthritis and 35 days for polyarthritis induced by the CFA.

### 5.3. Monoarthritis Induced by Zymosan

In this test, the protocol of Mbiantcha et al. [31] has been used with some modifications. One hour after oral administration of the various treatments, the thiopental is injected into each animal (0.1 ml/100 g, intraperitoneal route), and then zymosan A (0.3 ml, 0.9% v/v NaCl) was injected in the knee joint using a digital caliper (Mitutoyo, Japan); before the oral treatment, the thickness of the injected joint was measured, and then 1, 2, 3, 4, 5, 6, 24, 48, 72, 96, and 120 h after the injection of zymosan A. On the fifth day after taking the last parameters, the animals were again anesthetized, and the injected knee joint was incised and preserved in a formalin-PBS mixture (10%). The general scheme of method in histology was followed for histological analysis.

### 5.4. Polyarthritis Induced by CFA

The modified protocol of Mbiantcha et al. [40] was used. One hour after administration of each treatment, the animals were anesthetized by inhalation of ether vapor, and then 100 *μ*l of CFA (10 mg/ml) was injected into the tail vein, and then the animals were returned to well-labeled cages and observed.

The severity of arthritis was assessed by measuring the thickness of the hind leg joint using a digital caliper (days 0, 1, 3, 5, 7, 9, 11, 13, 15, 17, 19, 21, 23, 25, 27, 29, 31, 33, and 35), the pain sensitivity threshold using an analgesimeter (days 2, 4, 6, 8, 10, 12, 14, 16, 18, 20, 22, 24, 26, 28, 30, 32, and 34), and the arthritic score every day with certain criteria (score 0 = normal state, score 1 = edema on one foot or nodule, score 2 = two feet with edema or paw and nodule tail, score 3 = two feet with edema and nodosity of the tail or three feet with edema, and score 4 = three feet with edema and nodosity of tail or edema of four feet) and body weight every week.

At day 36, the animals were anesthetized with thiopental (0.1 ml/100 g body weight), the blood was removed by catheterization of the abdominal artery and introduced into two different types of tubes; tubes containing EDTA as anticoagulant for analysis of hematological parameters by standard laboratory method and tubes containing no anticoagulant were centrifuged (4900 rpm, 5 minutes), serum was collected to evaluate ALT, AST, ALP, MDA, creatinine, total protein, and stress parameters (NO, MAD, SOD, catalase, and glutathione). The weights of various organs such as liver, kidneys, thymus, and spleen were weighed, and knee joints of all animals were collected and stored in a formalin-PBS mixture (10%) for histological analysis.

### 5.5. Statistical Analysis

All *in vitro* test data indicate mean ± standard deviation in triplicate while for the *in vivo* test, the data are presented as an average of 6 animals ± SEM. Differences between groups were assessed by ANOVA (one way and two way) followed by Bonferroni posttest. Significant differences were considered at *p* < 0.05. *a*, *b*, and *c* or *α*, *β*, and *λ* denote significant differences with respect to the healthy control group and/or the arthritic control group.

## 6. Results

### 6.1. Chemical Composition

Several groups of chemical compounds have been demonstrated in extracts of *D. thollonii*. It can be seen from this table that the ethanolic extract contains all the test compounds with the exception of anthocyanins and triterpenes, whereas the aqueous extract contains only flavonoids, phenols, and polyphenols (Table 1).

## 7. *In* Vitro Anti-Inflammatory Activity

### 7.1. Inhibition of Protein Denaturation

For the results of this study, aqueous and ethanolic extracts effectively inhibit protein denaturation (albumin) caused by heat.  Table 2 shows significant inhibition (*p* < 0.001) of 42.51% and 44.44%, respectively, for the aqueous extract and the ethanolic extract at a concentration of 1000 *μ*g/ml, whereas diclofenac sodium produced 89.19% inhibition.

### 7.2. Cyclooxygenase Inhibitory Assay

Evaluation of cyclooxygenase activity determined the effect of both extracts on prostaglandin production. The results show that, at 1000 *μ*g/ml, the aqueous extract, the ethanolic extract, and ibuprofen significantly (*p* < 0.001) inhibit the activity of cyclooxygenase with 47.07%, 63.36%, and 97.88%, respectively (Table 2).

### 7.3. 5-Lipoxygenase Inhibitory Assay

The evaluation of the activity of 5-lipoxygenase was used to study the effect of the various extracts on the production of leukotrienes. This table shows that the aqueous and ethanolic extracts, as well as ibuprofen, have a significant inhibitory effect (*p* < 0.001) on the activity of 5-lipoxygenase with 66.79% inhibition, 77.48% and 95.31%, respectively (Table 2).

### 7.4. Effect of *D. thollonii* on Production of Intracellular ROS and T-Cell Proliferation

Table 3 presents the results for extracellular ROS production and T-cell proliferation. PMA is used to activate ROS and luminol as a developer. The ethanolic extract of *D. thollonii* significantly inhibited the release of ROS in whole blood with an IC_50_ of 4.98 *μ*g/ml. In the presence of polymorphonuclear, an inhibition was observed with an IC_50_ of 5. 74 *μ*g/ml and an IC_50_ of 2.96 *μ*g/ml with aqueous and ethanolic extracts. These extracts again inhibited the ROS produced by the macrophages with an IC_50_ of 7.47 *μ*g/ml for the aqueous extract and an IC_50_ of 3.28 *μ*g/ml for the ethanolic extract.

With respect to T-cell proliferation, both extracts showed significant antiproliferative property. Table 3 shows that the aqueous extract has an antiproliferative activity with an IC_50_ of 16.86 *μ*g/ml, while the ethanolic extract is 3.29 *μ*g/ml and that of prednisolone is less than 3.10 *μ*g/ml.

## 8. *In Vivo* Antiarthritis Assays

### 8.1. Effect of *D. thollonii* Treatment on Monoarthritis Induced by Zymosan A in Rats

**Figure 1:**
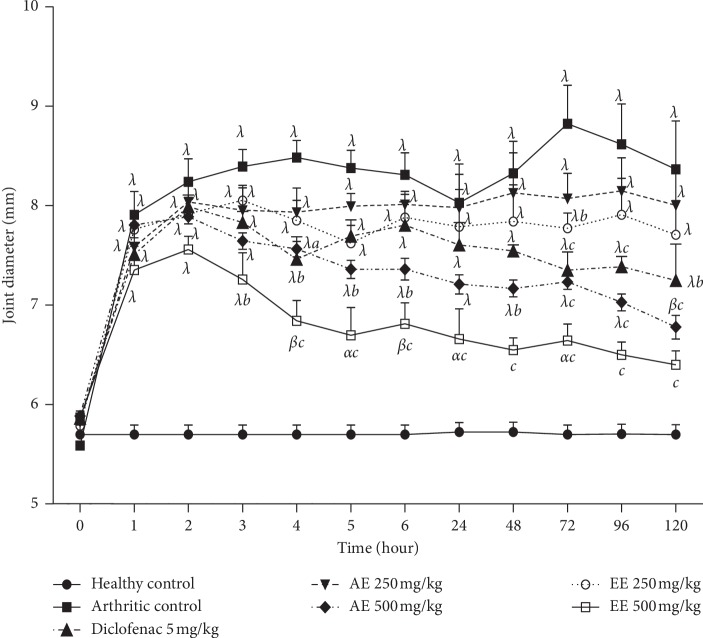
Effect of aqueous (AE) and ethanolic (EE) extracts of *Dissotis thollonii* on joint diameter in zymosan A-induced monoarthritis. Values are expressed as mean ± SEM for six animals and analyses by two-way ANOVA followed by Bonferroni post hoc test, ^*α*^*p* < 0.05, ^*β*^*p* < 0.01, and ^*λ*^*p* < 0.001 when compared with the healthy control and ^*a*^*p* < 0.05, ^*b*^*p* < 0.01, and ^*c*^*p* < 0.001 when compared with the arthritis control.

After injection of zymozan A in articulation of rats, knee joint results in the significant growth (*p* < 0.001) in the articular diameter, which is maximal from the first hour and still from the fifth day after the administration of zymosan A (Figure 1). Ethanolic and aqueous extracts of *D. thollonii* leaves (250 and 500 mg/kg) significantly (*p* < 0.05) inhibited zymosan A-induced edema. The inhibition of edema caused by the injection of the zymosan A was significant (*p* < 0.01) from the 3rd hour with a percentage inhibition of 52.90% for the ethanolic extract (500 mg/kg) and from the 4th hour with the aqueous extract (33.30%, *p* < 0.05, 500 mg/kg) and diclofenac (36.30%, *p* < 0.01, 5 mg/kg). The maximum and significant inhibitory effect (*p* < 0.001) was observed at the 5th day for the ethanolic extract (81.80%, 500 mg/kg), at the 5^th^ day for the aqueous extract (69.30%, 500 mg/kg), and at day 3 for diclofenac (53.10%, 5 mg/kg).

**Figure 2:**
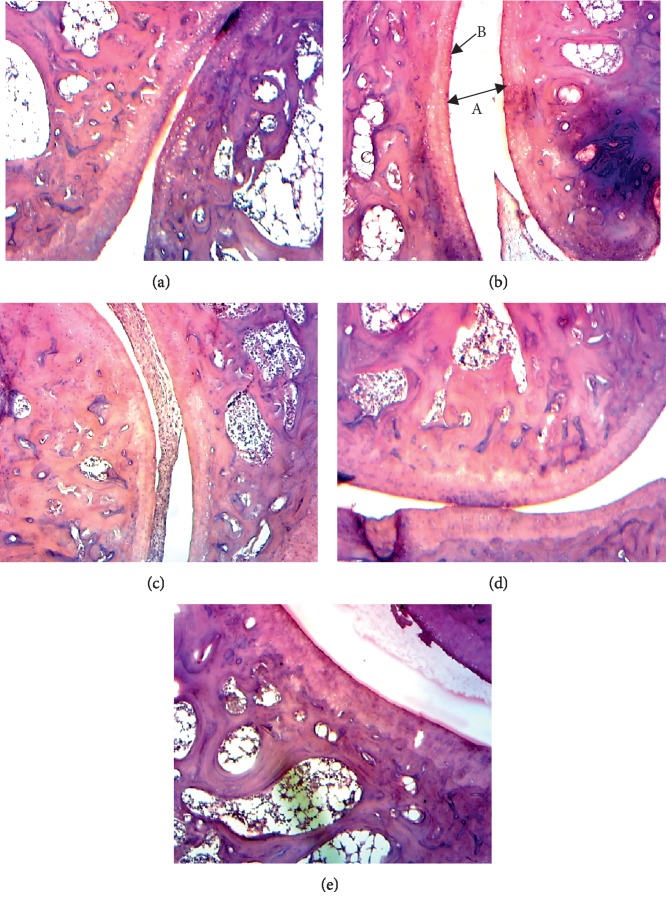
Histopathological analysis of ankle joints stained with H&E. (a) Healthy control showing normal structure with small joint space; (b) Arthritic control showing very large joint space (A), erosion of articular cartilage (B), and bone erosion (C), (c) diclofenac 5 mg/kg treated, (d) aqueous extract (AE) 500 mg/kg treated, and (e) ethanolic extract (EE) 500 mg/kg treated showing a decrease in joint space, erosion of the synovial membrane and a reduction of erosion of articular cartilage.

Figure 2 shows the histological analysis of the knee joints taken after injection of zymosan A. The architecture of the joint has a normal appearance in the nonarthritic control group; when in arthritic control, there is an erosion of the synovial membrane, a very large joint space, and erosion of bone and articular cartilage. However, treatment with aqueous and ethanolic extracts of *D. thollonii* prevented the destruction of the articular architecture of treated animals.

### 8.2. Effect of *D. thollonii* Treatment on Polyarthritis Induced by CFA in Rats

#### 8.2.1. Effect of *D. thollonii* Treatment on Joint Diameter

**Figure 3:**
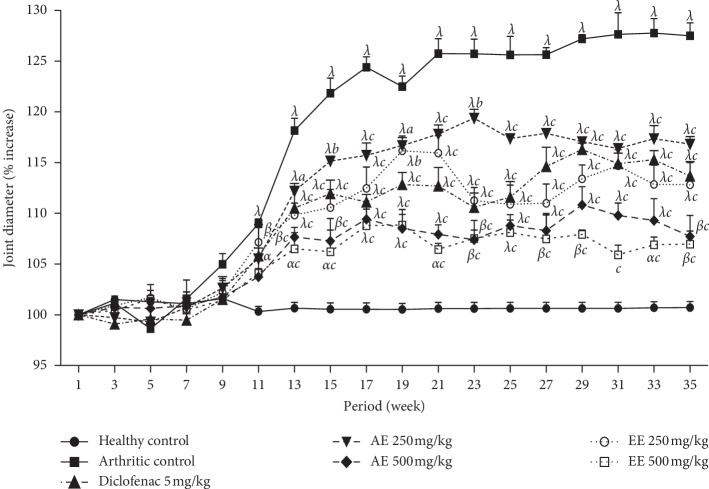
Effect of aqueous (AE) and ethanolic (EE) extracts of *Dissotis thollonii* on change in joint diameter in CFA-induced arthritis. Values are expressed as mean ± SEM for six animals and analyses by two-way ANOVA followed by *Bonferroni* post hoc test, ^*α*^*p* < 0.05, ^*β*^*p* < 0.01, and ^*λ*^*p* < 0.001 when compared with the healthy control and ^*a*^*p* < 0.05, ^*b*^*p* < 0.01, and ^*c*^*p* < 0.001 when compared with the arthritic control.

After the injection of CFA into the tail, the diameter of the joint increased significantly on the 11th day after injection (Figure 3). Aqueous and ethanolic extracts (500 mg/kg) bring out the significative (*p* < 0.001) reduction of joint diameter from day 13 with inhibition percentages of 54.34% and 65.48%, respectively, compared with the negative control. The inhibitory effect of different extracts remained significant during the days of experimentation. The maximum significant activity (*p* < 0.001) is observed at day 23 for diclofenac (60.03%, 5 mg/kg), at day 23 for the aqueous extract (71.85%, 500 mg/kg), and at the 31st day for the ethanolic extract (79.03%, 500 mg/kg).

#### 8.2.2. Effect of *D. thollonii* on Mechanical Nociceptive Pain Threshold

**Figure 4:**
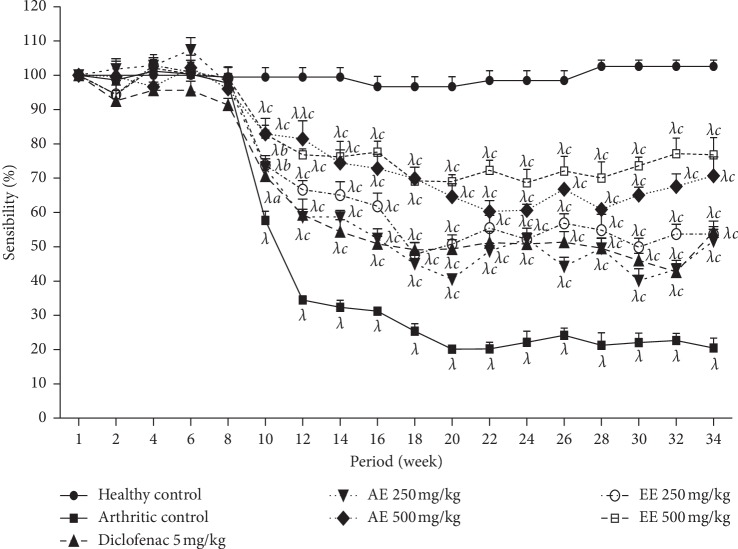
Effect of aqueous (AE) and methanolic (EE) extracts of *Dissotis thollonii* on mechanical hyperalgesia in CFA-induced arthritis. Values are expressed as mean ± SEM for six animals and analyses by two-way ANOVA followed by *Bonferroni* post hoc test, ^*β*^*p* < 0.01 and ^*λ*^*p* < 0.001 when compared with the healthy control and ^*a*^*p* < 0.05, ^*b*^*p* < 0.01, and ^*c*^*p* < 0.001 when compared with the arthritic control.

The induction of arthritis with CFA in rats generates the hypersensitivity threshold to mechanical pain which increased to day 10 still the end of experimentation (34^th^ day) with negative control. The continuous administration of different treatments significantly protected (*p* < 0.05, *p* < 0.01, and *p* < 0.001) animals against mechanical pain which was observed from the 10th day until the end of treatment. The maximal inhibitory activity is observed on the 12th day with the aqueous extract (500 mg/kg, *p* < 0.001, 69.00%), and on the 34th day with the ethanolic extract (500 mg/kg, *p* < 0.001, 70.35 %) and at day 34 with diclofenac (5 mg/kg, *p* < 0.001, 42.05 %). However, there is little improvement observed with the aqueous extract (250 mg/kg) with respect to mechanical pain sensitivity threshold (Figure 4).

#### 8.2.3. Effect of *D. thollonii* on Arthritic Score

**Figure 5:**
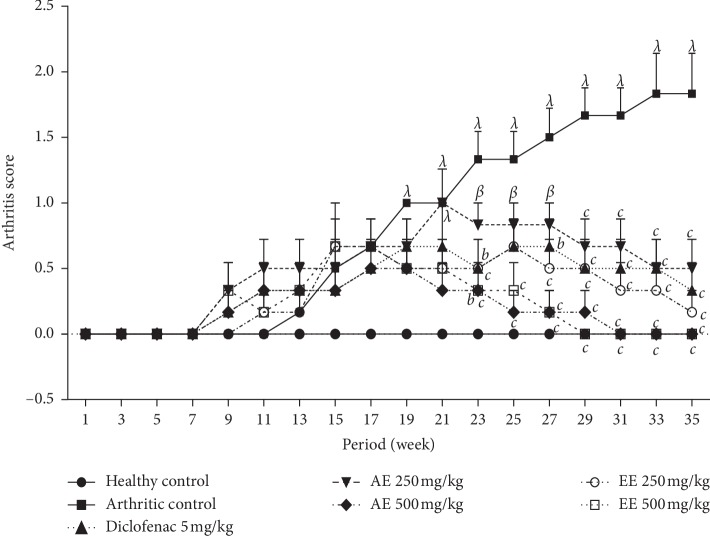
Effect of aqueous (AE) and ethanolic (EE) extracts of *Dissotis thollonii* on the arthritis score in CFA-induced arthritis. Values are expressed as mean ± SEM for six animals and analyses by two-way ANOVA followed by *Bonferroni* post hoc test, ^*λ*^*p* < 0.001 when compared with the healthy control and ^*b*^*p* < 0.01 and ^*c*^*p* < 0.001 when compared with the arthritic control.

The arthritic score does not materialize in normal controls, but in the groups that received CFA, it is very well materialized. Arthritic control groups are more affected from 11 until the end of treatment (Figure 5). Nevertheless, animals treated with different extracts showed a decrease in arthritic score values until the end of treatment. At the dose of 500 mg/kg, ethanolic extract has more activity than the aqueous extract because it has not only delayed the onset of arthritis materialization, but has also reduced the physical value of materialization. However, animals treated with diclofenac and ethanolic extract presented a significant (*p* < 0.001) reduction in the physical value of this materialization at the end of treatment.

#### 8.2.4. Effect of *D. thollonii* on Body Weight and Organ Weight

**Figure 6:**
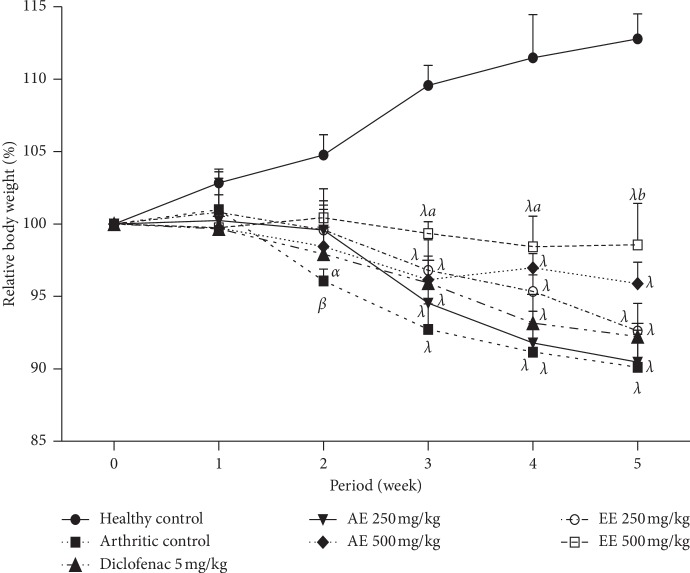
Effect of aqueous (AE) and ethanolic (EE) extracts of *Dissotis thollonii* on body weight in CFA-induced arthritis. Values are expressed as mean ± SEM for six animals and analyses by two-way ANOVA followed by Bonferroni post hoc test, ^*α*^*p* < 0.05, ^*β*^*p* < 0.01, and ^*λ*^*p* < 0.001 when compared with the healthy control and ^*a*^*p* < 0.05 and ^*b*^*p* < 0.01 when compared with the arthritic control.

In the arthritic control group, body weight decreased progressively and became significant (*p* < 0.01) from week 2 on positive group animals. In animals treated with the ethanolic extract (500 mg/kg), the change in body weight was significant (*p* < 0.05) at week 3 and persisted throughout treatment compared with arthritic control group animals. In animals from different groups treated with aqueous extract (500 and 250 mg/kg), with ethanolic extract (250 mg/mg), and with diclofenac (5 mg/kg), the change in body weight did not occur and was not significant (*p* < 0.05) throughout treatment (Figure 6).

**Figure 7:**
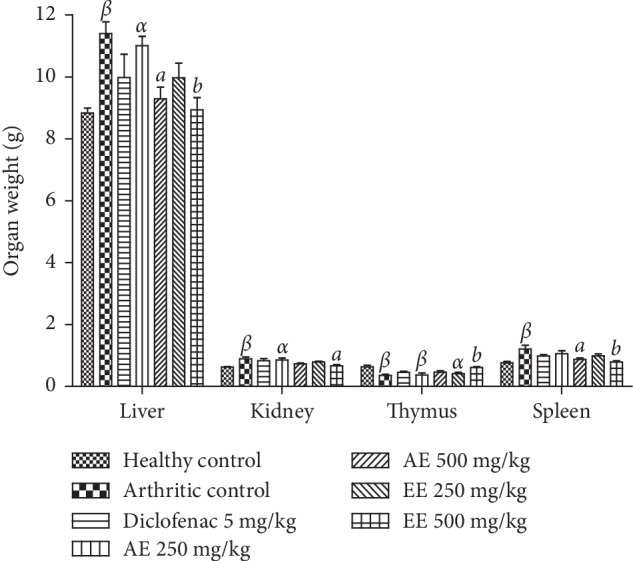
Effect of aqueous (AE) and ethanolic extracts (EE) of *Dissotis thollonii* on organ weight in CFA-induced arthritis. Values are expressed as mean ± SEM for six animals and analyses by one-way ANOVA followed by Bonferroni post hoc test, ^*α*^*p* < 0.05 and ^*β*^*p* < 0.01 when compared with the healthy control and ^*a*^*p* < 0.05 and ^*b*^*p* < 0.01 when compared with the arthritic control.

Results showed that weights of the liver, spleen, and kidney increased significantly (*p* < 0.01), and the weight of thymus decreased significantly (*p* < 0.01) in all control animals arthritic compared with the healthy control group. Recovery of organ weight balance was observed with continuous administration of the different extracts in arthritic animals compared with negative control (Figure 7).

#### 8.2.5. Effect of *D. thollonii* Extracts on Hematological Parameters

The results of the change in hematological parameters are shown in Table 4. In animals in the arthritic control group, platelet and WBC levels increased significantly (*p* < 0.001) in contrast to RBC, hemoglobin, and of hematocrit which decreased significantly (*p* < 0.001) compared with the healthy control. In treated animals with different doses of extracts (250 and 500 mg/kg) and diclofenac (5 mg/kg), the various parameters evaluated are close to those of animals in the healthy control group.

#### 8.2.6. Effect of *D. thollonii* Extracts on Biochemical Parameters

The biochemical parameters are shown in Table 5. Serum levels in arthritic animals show a significant (*p* < 0.001) increase in AST, ALT, ALP, and creatinine and a significant decrease (*p* < 0.001) of total protein relative to the serum level of the healthy control. These different levels improved considerably in animals treated with different extracts (aqueous and ethanolic) and diclofenac.

#### 8.2.7. Effect of *D. thollonii* Extracts on Oxidative Stress Parameters

The results on the change in oxidative stress parameters shown in Table 6 indicate that levels of NO and MDA increased significantly (*p* < 0.01) in arthritic animals, whereas levels of glutathione, catalase, and SOD decreased significantly (*p* < 0.01) compared with the healthy control. Animals treated with aqueous and ethanolic extracts, such as diclofenac, tend to improve these values.

#### 8.2.8. Histopathological Study

**Figure 8:**
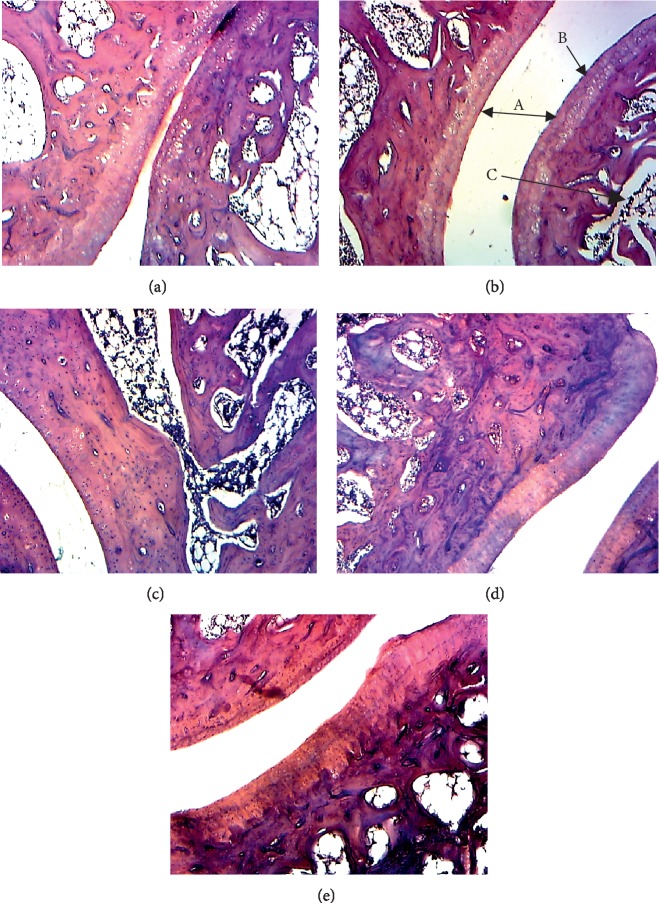
Histopathological analysis of ankle joints stained with H&E. (a) Healthy control showing normal structure with small joint space; (b) arthritic control showing very large joint space (A), destruction of cartilage (B), bone erosion (C), and cellular infiltration (D); (c) diclofenac 5 mg/kg treated, (d) aqueous extract (AE) 250 mg/kg treated, and (e) ethanolic extract (EE) 250 mg/kg treated showing a decrease in joint space, a reduction of cells infiltration and reduction of cartilage destruction.

A histopathological study of the knee joint showed no evidence of inflammation, bone erosion, cartilage destruction, or cellular infiltration in the healthy control. Animals in the arthritic control group presented joint architecture exhibiting cartilage destruction, bone erosion, and cellular infiltration; but animals treated with different extracts (250 and 500 mg/kg) and diclofenac (5 mg/kg) showed a significant protection of the architecture of the joint (Figure 8) by reducing bone erosion, cartilage destruction, and cellular infiltration.

## 9. Discussion

In the present study, the results show that aqueous and ethanolic extracts of *D. thollonii* have well anti-inflammatory properties *in vitro* in several models such as the inhibition of denaturation of proteins, 5-LOX, COX, and ROS. Inflammation, which is a very complex physiopathological response, involves the production of free radicals derived from neutrophils, NO, ROS, cytokines, and prostaglandins during its process [42]. Protein denaturation is the process by which proteins lose their tertiary structure and secondary structure. Proteins denaturation is a well-documented cause of inflammation [43]. The inflammation mechanism involves a series of events in which the metabolism of arachidonic acid plays an important role. Phenylbutazone, salicylic acid, flufenamic acid (anti-inflammatory drugs), etc., have shown a dose-dependent ability to thermally induced protein denaturation [44]. The pathogenesis of inflammatory diseases involves the overproduction of substances such as prostaglandin I2, thromboxane A2, prostaglandin E2, arachidonic acid, and leukotrienes through two metabolic pathways, the cyclooxygenase (COX) pathway and the 5-lipoxygenase (5-LOX) pathway [45]. In various inflammatory and allergic disorders, COX and 5-LOX are the main enzymes in the synthesis of prostanoids and eicosanoids from polyunsaturated fatty acids. The effective reduction of chronic inflammatory conditions is important by double inhibition of LOX and COX [46]. Substances capable of producing double inhibition of COX and 5-LOX with consequent substantial reduction in leukotriene and prostaglandin production produce a broad spectrum of anti-inflammatory activity and can be considered to have an excellent profile of pharmacological safety in clinical practice [47]. The anti-inflammatory activity of the aqueous and ethanolic extract of *D. thollonii* was determined using two methods, which were cyclooxygenase-2 (COX-2) and lipoxygenase (LOX) assays. All extracts tested showed significant activities vis-à-vis both tests. The inhibitory activity of aqueous and ethanolic extracts of *D. thollonii* on the denaturation of proteins, the cyclooxygenase and 5-lipoxygenase pathways, show that these two extracts are capable of significantly inhibiting the production of prostaglandins and leukotrienes, which gives these two extracts anti-inflammatory properties. These results are justified by the fact that compounds such as betulinic acid isolated from *D. thollonii* possess an in vitro inhibition property of cyclooxygenase (COX-1 and COX-2) and leukotriene B4 formation mediated by 5-LOX [48, 49].

The injection of zymosan into the knee joint causes a proliferative inflammatory monoarthritis resulting from the onset of an inflammatory reaction accompanied by hypernociception, an influx of neutrophils and leukocytes and then production by the infiltrated synovial cells and/or many inflammatory mediators include cytokines, free radicals, prostaglandins, and leukotrienes, which are responsible for the breakdown of articular cartilage, pannus formation, and synovial hypertrophy [50, 51]. Clinical management of arthritis is primarily aimed at reducing the intensity of pain, joint swelling, and then preventing or significantly reducing bone erosion and the various joint damages observed [52]. In this study, aqueous and ethanolic extracts of *D. thollonii* significantly reduced joint swelling up to the fifth hour of single administration, and the effect remains significant until the fifth day in continuous treatment. Similarly, the histological analysis of the joints has shown that the extracts of this plant, as well as diclofenac, induce a protective effect on the alteration of the synovial membrane, on bone erosion and considerably reduce cartilage lesions.

The caudal injection-induced polyarthritis of CFA in rats is a very good experimental model for preclinical evaluation of new antiarthritic agents; this model has many similarities with human rheumatoid diseases [53–55]. In addition, CFA injection induces hyperalgesia and allodynia by altering sensitivity to high-threshold nociceptor transduction [56]. In the present study, *D. thollonii* extracts showed an antiarthritic potential on all evaluated inflammatory parameters. These extracts significantly reduced the diameter of the joint in arthritic animals, and they significantly reduced the susceptibility of arthritic animals to mechanical pain, and they significantly improved the arthritic score. On these important parameters, the inhibitory effect of the extracts was significantly greater than that of diclofenac. In many disease states such as rheumatoid arthritis, decreased body weight is an important predictor of health [57]. The results of this study show that the extracts improved the body mass of the treated animals when compared with the untreated animals. During the development of rheumatoid arthritis, several enzymes are highlighted and represent good indices according to their importance (case of alkaline phosphatase and transaminases). The increase in serum levels of these enzymes would be implicated in periarticular osteopenia, in bone erosion and also indicate severe liver injury resulting in the production of biologically active substances in the inflammatory process [58–61]. In the present study, arthritic rat serum values ALP, AST, and ALT significantly increases, whereas in animals submited to different treatments (aqueous and ethanolic extracts of the leaves of *D. thollonii* or diclofenac) increased levels of these enzymes have been significantly reduced.

In the pathogenicity of rheumatoid arthritis, ROS, which are considered to be enhancers of inflammatory proliferation of the synovial membrane, play a key role in that their increased production increases the destruction of cartilage and even bones, activates or removes NF-*κ*B transcription factor, induces the production of many cytokines, and activates enzymes such as COX and 5-lipoxygenase or even inducible nitrogen monoxide [62–65]. The NF-*κ*B transcription factor is present in the cytosol of many cells where it is bound with the factor I-kB, especially those expressing cytokines, growth factors, chemokines, and adhesion molecules. Activation of the cell causes the phosphorylation of I-kB which is degraded followed by the release of the factor NF-*κ*B, which is introduced into the nucleus of the cell and causes the transcription of many proinflammatory mediators including iNOS, COX-2, and TNF-*α* and IL-1*β*, IL-6, and IL-8 [66]; thus, bone erosion and cartilage destruction observed in rheumatoid arthritis are due to overproduction of cytokines and inflammatory mediators. The aqueous and ethanolic extracts of the leaves of *D. thollonii* showed excellent antioxidant power, and in this study, the inhibition of the production of extracellular ROS in whole blood and in various phagocytic cells was significative (neutrophils and macrophages). This activity is thought to be due to the inhibition of pro inflammatory cytokine production (TNF alpha), the inhibition of denaturation of protein and the inhibition of the activity of proinflammatory enzymes such as COX and 5-lipoxygenase.

To investigate the effect of aqueous and ethanolic extracts of the leaves of *D. thollonii* on another aspect of the cellular immune response, the assay on T-cell proliferation was used. The results show that these *D. thollonii* extracts significantly inhibit T-cell proliferation, with a very significant result for the extracts compared with prednisolone used as a positive control. The results suggest that the compounds present in *D. thollonii* extracts are capable of significantly modulating, at different stages, the immune response. The significant decrease in the diameter of the joints observed macroscopically and histopathologically, followed by a decreased insensitivity to pain, clearly reveals the anti-inflammatory, antihyperalgic, and antiarthritic potential of *D. thollonii* extracts. It is possible that the effect of our different extracts is associated with an inhibition of the phosphorylation of the transcription factor NF-*κ*B. This is justified by the fact that compounds such as betulinic acid, ellagic acid, *β*-sitosterol, and ajuronic acid present in the extract of *D. thollonii* [30] have shown their ability to prevent degradation of the inhibitory I*κ*B-*α* protein, inducing inhibition of NF-*κ*B nuclear transcription factor activation, subsequent reduction of COX-2 expression, iNOS, inflammatory cell quantity, the levels of TNF-*α*, IL-6, IL-8, and increase the production of IL-10 [67–72]. Given that, during the development of the inflammatory reaction, the stimulation of the production of TNF-*α*, IL-1*β*, NO, and PGE2 would be linked to the activation of the NF-*κ*B/I*κ*B-*α* axis [73]; thus, the suppression of this axis has a significant therapeutic effect [72]. In addition, *D. thollonii* extracts showed in vitro inhibitory effects on TNF-*α* production, intracellular ROS production, leukocyte migration, and cell-proliferative HeLa cell line [24].

## 10. Conclusions

At the end of this study, the conclusion that we can deduce is that *D. thollonii* is a plant containing several groups of chemical compounds with anti-inflammatory and antiarthritic potential. These properties have been evaluated by *in vitro* and *in vivo* studies. In vitro studies have shown that *D. thollonii* has a very strong anti-inflammatory property: inhibition of protein denaturation, inhibition of 5-LOX, inhibition of COX and ROS and in vivo studies that have shown antiarthritis activity of the plant on a zymosan-induced monoarthritis model and a model of CFA-induced polyarthritis in the rat. These results confirm the utilization of this plant in the traditional treatment of chronic inflammatory diseases and consider it a potential candidate for the isolation of new anti-inflammatory and/or antiarthritic products.

## Figures and Tables

**Table 1:** Phytochemical profile of *Dissotis thollonii*.

Extracts	Phytochemical compounds								
	1	2	3	4	5	6	7	8	9
Aqueous extract	−	−	−	+	−	−	+	+	−
Ethanolic extract	−	+	+	+	-	+	+	+	−

−: absent; +: present; 1: triterpenoids; 2: sterols; 3: tannins; 4: flavonoids; 5: anthocyanings; 6: anthraquinones; 7: phenol/polyphenol; 8: saponins; 9: cardiac glycosides.

**Table 2:** Effect of aqueous and ethanolic extracts of *Dissotis thollonii* on protein denaturation, cyclooxygenase inhibition, and 5-lipoxygenase inhibition.

Compounds	Dose (*μ*g/ml)	Inhibition (%)		
		Protein denaturation	Cyclooxygénase	5-lipoxygénase
Diclofenac	100	74.41	—	—
	200	76.72	—	—
	500	81.48	—	—
	1000	89.19	—	—
Ibuprofen	100	—	83.46	82.03
	200	—	89.90	88.63
	500	—	93.39	92.96
	1000	—	97.88	95.31
Aqueous extract	100	27.33	24.72	31.26
	200	29.26	34.14	45.60
	500	33.83	40.70	58.17
	1000	42.51	47.07	66.79
Ethanolic extract	100	30.87	28.14	36.12
	200	32.79	40.89	48.24
	500	37.30	53.55	64.11
	1000	44.44	63.36	77.48

The percentage values were obtained using various concentrations of test compounds, and readings are presented as mean of triplicates.

**Table 3:** IC_50_ value of aqueous and ethanolic extracts of *Dissotis thollonii* on human whole blood evaluated by luminal-amplified chemiluminescence.

	Oxidative burst (IC_50_ (*μ*g/ml))			T-cell proliferation (IC_50_ (*μ*g/ml))
	WB	PMNs	MQ	
Aqueous extract	>100	5.74 ± 0.65	7.47 ± 2.68	16.89 ± 2.55
Ethanolic extract	4.89 ± 0.49	2.96 ± 0.12	3.28 ± 0.09	3.29 ± 1.96
Ibuprofen	12.71 ± 0.13	12.53 ± 0.21	13.28 ± 1.07	—
Prednisolone	—	—	—	<3.10

The IC_50_ values were obtained using various concentrations of test compounds, and readings are presented as mean ± SD of triplicates. WB: whole blood; PMNs: polymorphonuclear leukocytes; MQ: mice peritoneal macrophages.

**Table 4:** Influence of the aqueous and ethanolic extracts of *Dissotis thollonii* on haematological in CFA-induced arthritis in rats.

Treatment	Dose (mg/kg)	Haemoglobin (g/dl)	Hematocrit (%)	Platelet (10^9^/L)	RBC (million/*μ*l)	WBC (10^9^/L)
Healthy control	—	16.40 ± 0.73	38.78 ± 1.03	365.25 ± 26.23	7.22 ± 0.34	7.85 ± 0.44
Arthritic control	—	9.43 ± 0.20^*λ*^	25.65 ± 1.27^*λ*^	554.75 ± 21.54^*λ*^	3.90 ± 0.37^*λ*^	13.78 ± 0.56^*λ*^
Diclofenac	5	14.15 ± 0.62^*b*^	33.88 ± 2.59^*a*^	481.75 ± 13.31^*β*^	5.65 ± 0.24^*a*^	10.63 ± 0.64^*αa*^
Aqueous extract	250	14.28 ± 0.67^*b*^	31.15 ± 1.23^*α*^	478.74 ± 18.71^*β*^	5.74 ± 0.40^*a*^	11.85 ± 0.80^*λ*^
	500	15.55 ± 0.59^*c*^	37.73 ± 1.28^*c*^	383.50 ± 9.75^*c*^	6.93 ± 0.25^*c*^	10.25 ± 0.41^*b*^
Ethanolic extract	250	14.33 ± 1.02^*b*^	32.20 ± 1.70	426.00 ± 18.11^*c*^	6.31 ± 0.38^*c*^	11.15 ± 0.48^*β*^
	500	16.88 ± 1.32^*c*^	38.38 ± 0.99^*c*^	374.75 ± 8.28^*c*^	7.36 ± 0.29^*c*^	8.28 ± 0.42^*c*^

CFA: complete Freund's adjuvant; RBC: red blood cell; WBC: white blood cell. Each value represents the mean ± ESM of six animals. ^*α*^*p* < 0.05; ^*β*^*p* < 0.01; ^*λ*^*p* < 0.001 statistically significant compared with the healthy control. Each value represents the mean ± ESM of 6 animals. ^*a*^*p* < 0.05; ^*b*^*p* < 0.01; ^*c*^*p* < 0.001 statistically significant compared to arthritic control.

**Table 5:** Effect of aqueous and ethanolic extracts of *Dissotis thollonii* on serum parameters in CFA-induced arthritis in rats.

Treatment	Dose (mg/kg)	ALT (U/I)	AST (U/I)	ALP (U/I)	Creatinine (*μ*mol/l)	Total protein (g/dl)
Healthy control	—	47.09 ± 1.91	173.89 ± 11.61	90.27 ± 3.36	0.48 ± 0.07	7.45 ± 0.24
Arthritic control	—	77.88 ± 2.07^*λ*^	423.09 ± 23.51^*λ*^	444.38 ± 13.69^*λ*^	1.39 ± 0.13^*λ*^	5.90 ± 0.17^*β*^
Diclofenac	5	63.46 ± 1.50^*λc*^	250.70 ± 16.76^*βc*^	240.11 ± 16.28^*λc*^	0.93 ± 0.03^*λc*^	6.65 ± 0.28
Aqueous extract	250	63.97 ± 1.72^*λc*^	299.79 ± 11.76^*λc*^	317.40 ± 17.20^*λc*^	0.98 ± 0.05^*λc*^	6.35 ± 0.34
	500	55.14 ± 1.16^*αc*^	222.59 ± 6.07^c^	207.33 ± 9.89^*λc*^	0.69 ± 0.03^*c*^	7.31 ± 0.19^*a*^
Ethanolic extract	250	55.41 ± 1.84^*αc*^	253.30 ± 2.77^*βc*^	270.97 ± 7.43^*λc*^	0.86 ± 0.3^*βc*^	7.16 ± 0.29^*a*^
	500	46.12 ± 1.42^*c*^	177.62 ± 7.62^*c*^	143.00 ± 5.34^*c*^	0.60 ± 0.02^*c*^	7.54 ± 0.28^*b*^

CFA: complete Freund's adjuvant; ALP: alkaline phosphatase; AST: aminotransferase; ALT: alanine aminotransferase. Each value represents the mean ± ESM for six animals and analyses by one-way ANOVA followed by Bonferroni post hoc test, ^*α*^*p* < 0.05, ^*β*^*p* < 0.01, ^*λ*^*p* < 0.001 when compared with the healthy control and ^*a*^*p* < 0.05, ^*b*^*p* < 0.01, ^*c*^*p* < 0.001 when compared with the arthritic control.

**Table 6:** Effect of aqueous and ethanolic extracts of *Dissotis thollonii* on some parameters of oxidative stress in CFA-induced arthritis in rats.

Treatment	Dose (mg/kg)	NO (*μ*M)	Glutathion (×10^3^ activity)	Catalase (activity)	MDA (×10^3^ mol/l)	SOD (activity)
Healthy control	—	0.048 ± 0.003	0.28 ± 0.03	52.99 ± 0.67	0.087 ± 0.021	1.93 ± 0.02
Arthritic control	—	0.089 ± 0.003^*λ*^	0.17 ± 0.01^*β*^	36.89 ± 0.72^*λ*^	0.138 ± 0.027	1.51 ± 0.03^*λ*^
Diclofenac	5	0.044 ± 0.001^*c*^	0.21 ± 0.01	37.72 ± 0.74^*γ*^	0.112 ± 0.035	1.92 ± 0.03^*c*^
Aqueous extract	250	0.073 ± 0.005^*λa*^	0.18 ± 0.02^*β*^	45.33 ± 0.97	0.127 ± 0.020	1.93 ± 0.02^*c*^
	500	0.064 ± 0.003^*αc*^	0.24 ± 0.01	51.45 ± 1.70^*c*^	0.109 ± 0.038	1.95 ± 0.04^*c*^
Ethanolic extract	250	0.067 ± 0.001^*βc*^	0.22 ± 0.01	48.95 ± 1.18^*c*^	0.125 ± 0.005	1.94 ± 0.02^*c*^
	500	0.046 ± 0.003^*c*^	0.27 ± 0.01^*b*^	52.39 ± 4.09^*c*^	0.071 ± 0.020	1.95 ± 0.08^*c*^

Each value represents the mean ± ESM for six animals and analyses by one-way ANOVA followed by Bonferroni post hoc test, ^*α*^*p* < 0.05, ^*β*^*p* < 0.01, and ^*λ*^*p* < 0.001 when compared with the healthy control and ^*a*^*p* < 0.05, ^*c*^*p* < 0.001 when compared with the arthritic control.

## Data Availability

All data supporting our findings are adequately contained within the manuscript.
